# Detection of Methylphenidate in Equine Hair Using Liquid Chromatography–High-Resolution Mass Spectrometry

**DOI:** 10.3390/molecules26195798

**Published:** 2021-09-24

**Authors:** Benjamin C. Moeller, Luis Flores, Amel Clifford, Gwendolyne Alarcio, Mary Mosburg, Rick M. Arthur

**Affiliations:** 1 KL Maddy Equine Analytical Chemistry Laboratory, School of Veterinary Medicine, University of California, Davis, CA 95616, USA; laflore@ucdavis.edu (L.F.); amlclifford@ucdavis.edu (A.C.); gsgonzales@ucdavis.edu (G.A.); mmmosburg@ucdavis.edu (M.M.); 2 Department of Molecular Biosciences, School of Veterinary Medicine, University of California, Davis, CA 95616, USA; 3 School of Veterinary Medicine, University of California, Davis, CA 95616, USA; rmarthur@ucdavis.edu

**Keywords:** methylphenidate, horse, liquid chromatography–mass spectrometry, hair, anti-doping

## Abstract

Methylphenidate is a powerful central nervous system stimulant with a high potential for abuse in horse racing. The detection of methylphenidate use is of interest to horse racing authorities for both prior to and during competition. The use of hair as an alternative sampling matrix for equine anti-doping has increased as the number of detectable compounds has expanded. Our laboratory developed a liquid chromatography–high-resolution mass spectrometry method to detect the presence of methylphenidate in submitted samples. Briefly, hair was decontaminated, cut, and pulverized prior to liquid–liquid extraction in basic conditions before introduction to the LC-MS system. Instrumental analysis was conducted using a Thermo Q Exactive mass spectrometer using parallel reaction monitoring using a stepped collision energy to obtain sufficient product ions for qualitative identification. The method was validated and limits of quantitation, linearity, matrix effects, recovery, accuracy, and precision were determined. The method has been applied to confirm the presence of methylphenidate in official samples submitted by racing authorities.

## 1. Introduction

Methylphenidate is a synthetic central nervous system stimulant frequently prescribed to treat attention deficit disorder and narcolepsy in humans [1]. Methylphenidate is a prohibited substance in horseracing and is classified as a Class 1 A drug by the Association of Racing Commissioners International and as an S6 prohibited substance by the World Anti-Doping Association [2]. Given its high potential for abuse and risk for dependence, it is a Schedule II drug by the US Drug Enforcement Agency. Methylphenidate was first synthesized in the 1940s, approved by the US FDA in the mid-1950s, and marketed by its brand name Ritalin. Shortly afterwards, concerns about its performance-enhancing effects on the horse began to be raised [1,3]. 

Studies conducted in the 1960s clearly show that methylphenidate administration to horses at doses ranging from ≈0.1 to 1.2 mg/kg have effects on the central nervous system with increases in pulse, respiratory rate, and blood pressure observed [3]. The absorption, distribution, metabolism, and excretion properties of methylphenidate or structurally similar agents such as ethylphenidate have not been investigated in great detail in the horse, but some studies have shown that these compounds are relatively quickly cleared and primarily excreted in urine following intramuscular, intravenous, or oral administrations [3,4,5]. The major metabolite of methylphenidate in humans is ritalinic acid. Studies with incubations of methylphenidate in equine liver microsomes confirm that ritalinic acid along with mono- or di-hydroxylations of either methylphenidate or ritalinic acid are capable of being formed [6]. 

The detection and confirmation of methylphenidate in urine and blood samples is challenging due to its rapid distribution and elimination half-lives, its metabolism to ritalinic acid, and the higher background of its major product ion (84 *m*/*z*), which makes low-level detection challenging [3,4,5]. Analysis of hair samples offers an alternative sampling matrix that can extend the potential detection window following the administration of a prohibited substance [7]. There have been several reports of the detection of methylphenidate in the hair of humans associated with either therapeutic use or abuse of the compound using either gas chromatography–mass spectrometry (GC-MS) or liquid chromatography–mass spectrometry (LC-MS) [8,9,10,11,12]. The increased sensitivity afforded by modern LC-MS equipment as compared to older GC-MS-based technology has made low-level detections (≈0.5 pg/mg) of methylphenidate possible, although many investigators only monitored the presence of a single product ion (84 *m/z*). The formation of sufficient product ions required for definitive qualitative identification is challenging for methylphenidate, as a single product ion dominates the fragmentation spectra. This is particularly pronounced when using full-scan MS/MS fragmentation as opposite to spectra generated using selected reaction monitoring (SRM) on a triple quadrupole mass spectrometer, as one typically chooses a single collision energy for fragmentation when acquiring full-scan MS/MS spectra as opposed to different collision energies being applied for each transition when using SRM. To the best of the authors’ knowledge, there are no reports of methods capable of detecting methylphenidate use in equine hair.

Most anti-doping laboratories screen for methylphenidate and/or ritalinic acid as part of their routine testing. In spite of this routine testing, there have been continued suspicions of the abuse of methylphenidate in horse racing for horses both in training and during competition. To address these concerns, our laboratory recently incorporated screening for methylphenidate in our out-of-competition hair-testing paradigm and identified several samples as suspects potentially containing methylphenidate. This paper describes the development and validation of a targeted LC-MS approach using a high-resolution accurate mass (HRMS) instrument to confirm the presence of methylphenidate in equine hair and its application to official out-of-competition samples.

## 2. Results

**Figure 1:**
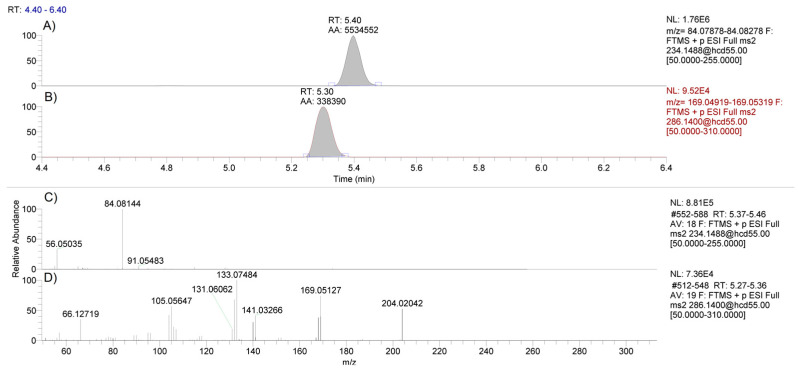
LC-MS chromatograms of methylphenidate using the 234.1488 *m/z* precursor ion (panel **A**) and the internal standard D9-clenbuterol using the 286.1400 *m/z* precursor ion (panel **B**). PRM spectra for methylphenidate (panel **C**) and D9-clenbuterol (panel **D**) following HCD fragmentation using a stepped collision energy of 30 and 80 eV in a standard.

A fit-for-purpose LC-MS method for the detection and confirmation of methylphenidate and D9-clenbuterol (internal standard) in equine hair was developed and validated using liquid–liquid extraction at a basic pH and analysis via LC-HRMS. Using parallel reaction monitoring (PRM) scans, the method was highly sensitive and selective for methylphenidate and the internal standard, D9-clenbuterol. Good chromatographic performance of both compounds was achieved, and the formation of three product ions (56.04948, 84.08078, 91.05423 *m*/*z*) of methylphenidate with relative abundances above 5% following higher-energy C-trap dissociation (HCD) using a stepped collision energy at 30 and 80 eV (Figure 1) allows for unambiguous qualitative determination.

**Figure 2:**
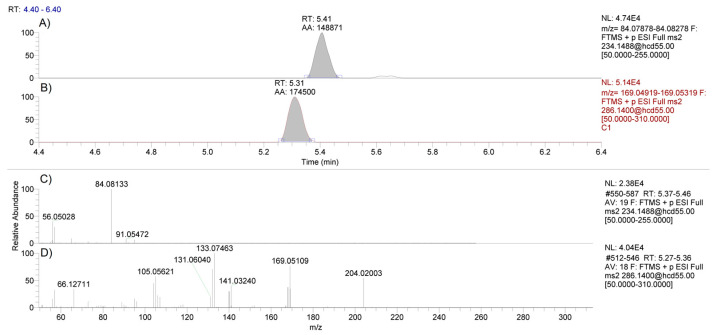
LC-MS chromatograms of methylphenidate using the 234.1488 *m/z* precursor ion (panel **A**) and the internal standard D9-clenbuterol using the 286.1400 *m/z* precursor ion (panel **B**). PRM spectra for methylphenidate (panel **C**) and D9-clenbuterol (panel **D**) following HCD fragmentation using a stepped collision energy of 30 and 80 eV in a hair sample spiked with methylphenidate at 1 pg/mg.

The limit of detection was determined to be 0.3 pg/mg, and the limit of quantification of methylphenidate in spiked hair was 1.0 pg/mg (Figure 2). The concentration of methylphenidate was determined using the peak area ratio of methylphenidate to its internal standard and linear regression analysis with 1/x weighting applied. The linear range was determined to be 1 to 40 pg/mg with regression correlation coefficients, r^2^, of 0.980 or higher across multiple runs (Figure S1). 

**Figure 3:**
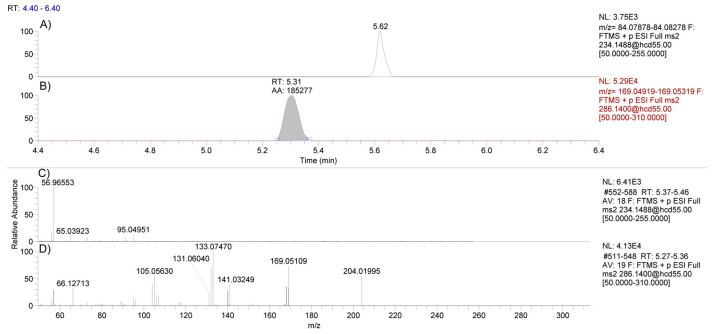
LC-MS chromatograms of methylphenidate using the 234.1488 *m/z* precursor ion (panel **A**) and the internal standard D9-clenbuterol using the 286.1400 *m/z* precursor ion (panel **B**). PRM spectra for methylphenidate (panel **C**) and D9-clenbuterol (Panel **D**) following HCD fragmentation using a stepped collision energy of 30 and 80 eV in a negative control hair sample.

The specificity of the method was assessed during the validation period, and no methylphenidate was detected in the extracted ion chromatograms of negative control samples at the corresponding retention time for methylphenidate (Figure 3). The inter- and intra-day accuracy and precision of the method was determined over 3 days of validation with accuracy (range of ≈82–99% expected) and precision (range of ≈3–19 relative standard deviation), showing acceptable results (Table 1).

Recovery (26% for methylphenidate and 99% for D9-clenbuterol) was determined by comparing the peak area of samples (*n* = 6) spiked before extraction and after extraction at 10 pg/mg methylphenidate and D9-clenbuterol. Matrix effects were evaluated at 10 pg/mg level by dividing the peak areas of both compounds from post extraction spiked samples by the peak areas of neat standards, which were prepared at equivalent concentrations. The matrix effects were determined to be 0.7 and 0.6 for methylphenidate and D9-clenbuterol, respectively.

**Figure 4:**
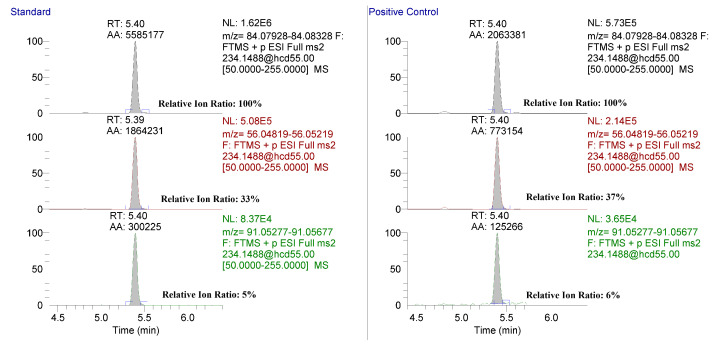
Extracted ion chromatograms for the targeted product ions from a PRM scan of methylphenidate in a neat standard (**left**) and a positive control hair sample spiked with methylphenidate at 10 pg/mg (**right**). Relative ion ratios for both the standard and spiked sample are shown.

Association of Official Racing Chemist (AORC) qualitative identification criteria were applied to identify methylphenidate using the retention time, product ion ratios, and accurate mass criteria [13]. The retention time of methylphenidate was 5.4 min and 5.3 min for D9-clenbuterol and was highly reproducible within each analytical run. Extracted ion chromatograms for the 56.04948, 84.08078, and 91.05423 *m*/*z* product ions from neat standards were compared to those of a hair sample spiked with methylphenidate at 10 pg/mg (Figure 4). The relative ion ratios and retention time met the AORC identification criteria for hair samples spiked at 1 to 40 pg/mg across the validation runs (data not shown). A 2 mDa mass window was used in generating extracted ion ratios in compliance with AORC identification criteria. 

**Figure 5:**
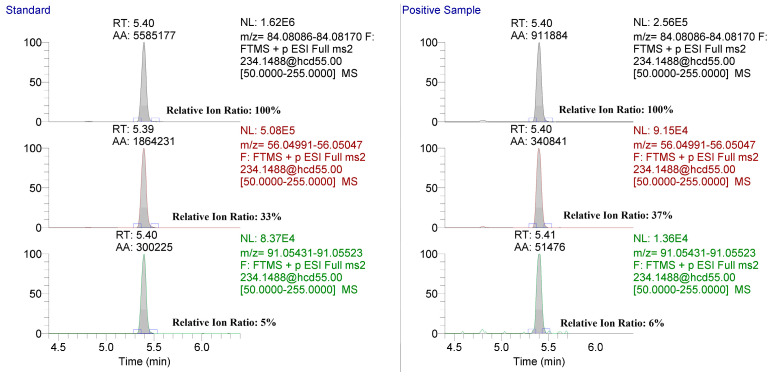
Extracted ion chromatograms for the targeted product ions from a PRM scan of methylphenidate in a neat standard (**left**) and an out-of-competition hair sample submitted for analysis (**right**). Relative ion ratios for both the standard and out-of-competition sample are shown.

After validation, the method was applied to confirm the presence of methylphenidate in official samples submitted to the laboratory following LC-MS based screening. Extracted ion chromatograms for 56.04948, 84.08078, and 91.05423 *m*/*z* product ions from a standard and an official out-of-competition hair sample are shown Figure 5. The relative ion ratios and retention times met the AORC identification criteria [13].

## 3. Discussion

The use of hair testing in equine anti-doping represents an additional alternative sampling matrix that may offer additional coverage for some compounds and can be a useful approach to supplement traditional urine/blood-based testing [7,14,15]. The ability to detect compounds weeks to months after administration greatly expands the coverage window for some compounds. As equine hair on the mane and tail grows at ≈2–3 cm per month, this offers a reproducible sampling location with the ability to have repeated sampling occur from the sample animal at various time points [16,17]. The exact mechanism for incorporation of various drugs, including methylphenidate into hair is not fully understood, but in general, basic compounds more readily partition into hair as compared to acidic compounds [7,9,18,19]. Prior to implementing hair testing into official samples, one should consider the length of hair collected and length of segments (if desired), which influences the time period of drug coverage. Additionally, while the results reported in this manuscript provide quantitative results, some caution is warranted as the extraction efficiency and recovery of a compound from a hair sample from actual administrations may not fully reflect those of a hair sample that has been spiked or soaked with that same compound.

In human sport, the World Anti-Doping Association has not incorporated hair testing as a valid primary specimen for doping control, although it has been used to supplement investigations of adverse analytical findings. There have been many recent investigations into the use of hair as an alternative sampling matrix for a number of doping compounds, and the applicability of hair testing in human anti-doping has been reviewed by several authors [7,15,18,20]. As the use of hair testing for prohibited substances in both equine and human anti-doping applications expands, further considerations into the potential routes of exposure, stability of compounds after incorporation, rates of incorporation of compounds into hair, and the minimum dose sufficient to be detected in a hair sample will be explored in more detail. 

There are several reports of the detection of methylphenidate in human hair summarized in Table 2, but to the best of the author’s knowledge, this is the first report in the horse [8,10,11,12]. The majority of reports have utilized LC-MS-based analysis, although GC-MS analysis has also been reported. Koster et al. utilized LC-MS analysis as part of a multi-analyte screening method using liquid–liquid extraction with an LOD of 30 pg/mg [12]. Marchei et al. utilized LC-MS-based analysis following extraction using a solid phase extraction cartridge with an LOD of 50 pg/mg [11]. Sticht et al. utilized GC-MS analysis following liquid–liquid extraction using isohexane in basic conditions to achieve an LOD of 20 pg/mg [10]. While most investigations have focused on the detection of methylphenidate, recently, Jang et al. utilized LC-MS analysis with a methanolic extraction approach to target both methylphenidate and ritalinic acid with LODs of 0.5 and 1.0 pg/mg, respectively [8]. Analysis of hair from exposures in humans showed that methylphenidate was consistently present at ≈2–5× higher concentrations as compared to ritalinic acid [8]. Given that methylphenidate is metabolized to ritalinic acid, which can be found at higher concentrations in blood and urine as compared to the parent drug, this ratio suggests that ritalinic acid poorly incorporates into hair. This is not surprising, as it is well-established that a number of factors influence the incorporation of compounds into hair, generally with polar acidic molecules being found at lower levels as compared to non-polar basic compounds [7,8]. 

In general, LC-MS analysis for methylphenidate is highly sensitive with detection limits considerably lower as compared to GC-MS. Our reported limit of quantification (1 pg/mg) is in line with those observed by Jang et al. using a modern triple quadrupole mass spectrometer and ≈20–50× lower than those reported by others [8]. With LC-MS analysis, particularly using an instrument capable of MS/MS affords high sensitivity, the approach is limited as a single product ion (84 *m/z*) as the major fragment using collision-induced dissociation or similar fragmentation approaches [8,11,12]. The lack of high abundance product ions makes low-level confirmation of methylphenidate challenging. GC-MS analysis, following derivatization, offers a larger number of product ions, which is useful in reporting positive findings in accordance with AORC criteria, but the methodology does not have the necessary sensitivity to confirm the presence of methylphenidate at low levels [10]. Our approach of a stepped collision energy (30, 80 eV) with HCD fragmentation prior to introduction into the Orbitrap on the Q-Exactive system allows for the detection of at least three product ions with good relative abundances (Figure 4). The use of HRMS in our method allows for the unambiguous detection of product ions with minimal background interferences noted. 

There are some limitations of our approach that should be noted. First, in future studies, the use of stable isotope-labeled clenbuterol as the internal standard can be replaced with stable isotope-labeled methylphenidate, which should improve the method’s quantitative performance. Secondly, the incorporation of the ability to detect ritialinic acid in our methodology would strengthen the methodology and allow for further verification of methylphenidate exposures. Unfortunately, this was not directly assessed in our method, and the extraction conditions utilized in our approach may not allow for as good of recovery as compared to the methanolic extracts used by Jang et al. [8]. Our use of an acidic distribution step prior to pH adjustment to basic conditions before extraction may be modified to more simple approaches as shown by Jang et al., but this was not directly assessed in our study. Additionally, given the low levels of methylphenidate found in submitted anti-doping samples, further improvements in detection limits may identify the abuse of this agent at levels our below our current methodological capabilities. 

Methylphenidate is a Schedule II US DEA controlled substance due to its high potential for human abuse. There is no recognized use in veterinary medicine, but methylphenidate has a history of abuse in Quarter Horse sprint racing and unsanctioned match racing, which are usually sprints. Our laboratory recently incorporated methylphenidate into routine screening procedures for hair sample submitted to the laboratory for analysis, and we detected several suspect findings shortly thereafter that were confirmed (≈1–6 pg/mg range) using this methodology. It should be noted that while we generated estimated concentrations, all positive findings were reported as qualitative results. Out of over 600 samples analyzed, all positives were obtained from Quarter Horses and none in Thoroughbred horse racing.

The pharmacological effects of methylphenidate on both the central nervous and cardiovascular systems make it a compound that should be regulated during competition, and thus, it is commonly tested for in routine post-race drug testing. As individuals are aware of the testing for horses actively competing, it is believed that methylphenidate, at least in the racing jurisdictions in which our laboratory provides testing, is primarily utilized as a training aide for horses that receive stimulatory effects either in training events or during timed qualifying exercise sessions. During the course of investigating positive cases, there were medical records recording the administration of 30 mg of methylphenidate approximately 2–4 months prior to sample collection. This dose (≈0.06 mg/kg) is in-line with the low end of the dose range (0.1–1 mg/kg) utilized in administration studies ≈40–60 years ago [3,5]. There have been reports of concentrations of methylphenidate in the ≈150–4000 pg/mg range in hair from known chronic use (≈5–40 mg methylphenidate per day) in the treatment of attention deficit disorder in children [11]. As the likely pattern of abuse of methylphenidate in horseracing is at much less frequent administrations as compared to these reports, one should expect relatively lower concentrations of methylphenidate being present in the hair of treated horses.

## 4. Materials and Methods

### 4.1. Chemicals and Reagents

Methylphenidate (1 mg/mL) was purchased from Cerilliant (Round Rock, TX, USA). D9-Clenbuterol (0.2 mg/mL) was purchased from Frontier Biopharm (Richmond, KY, USA). Working solutions of methylphenidate and D9-clenbuterol were made at various concentrations in methanol. Formic acid was purchased from EM Science (Gibbstown, NJ, USA). Water and acetonitrile were purchased from Burdick & Jackson (Muskegon, MI, USA). Sodium hydroxide and ethyl acetate were purchased from Fisher Scientific (Waltham, MA, USA). Negative control hair was obtained from University-owned animals in accordance with approved Institutional Animal Care and Use Committee authorization. 

### 4.2. Sample Preparation

Equine hair (≈10 mm section) was decontaminated by washes with deionized water prior to drying at 40 °C for 30–45 min. After drying, the hair was cut into smaller sections (≈2–4 mm) and pulverized using an Omni Bead Mill (Kennesaw, GA, USA) homogenizer. The pulverized hair (50 mg) was transferred to a glass vial and spiked with 50 µL of the internal standard (10 pg/µL) in methanol and allowed to soak for ≈20 min. After the addition of the internal standard, 1.5 mL of 0.1 M acetic acid was added, and samples were incubated for 30 min at 65 °C. After disruption of the hair, 3 mL of 0.1 M sodium hydroxide was added, and the pH was adjusted to 12 with 2N sodium hydroxide (if necessary). Then, the samples were centrifuged for 5 min at 3000 rpm, and the supernatant was removed and combined with 5 mL of ethyl acetate. After the addition of ethyl acetate, the samples were rotated for 10 min prior to centrifugation for 5 min at 3000 rpm. After centrifugation, the ethyl acetate was removed to a 12 mm × 75 mm glass vial and evaporated using nitrogen in a Turbovap at 45 °C. Then, extracts were reconstituted in 120 µL of 95/5% water/acetonitrile with 0.2% formic acid and transferred to autosampler vials with inserts prior to introduction to the LC-MS system.

### 4.3. Calibration and Quality Control Samples

Seven calibration samples ranging from 1 to 40 pg/mg of methylphenidate were prepared. Three quality control (QC) levels were prepared at 3, 10, and 30 pg/mg. 

### 4.4. Method Validation

The method was validated as a fit-for-purpose method to confirm methylphenidate in equine hair. The following parameters were monitored: limit of detection, limit of quantitation, linear range, recovery, matrix effects, accuracy and precision, and qualitative identification per AORC criteria [13]. The limit of detection was calculated using the equation, LOD = response from blanks + 1.645 (Standard Deviation of low QC) [21]. Accuracy (% accuracy) and precision (relative standard deviation) were assessed at each QC level (*n* = 6/level) across 3 days. Peak areas for methylphenidate and D9-clenbuterol from neat standards, extracted samples spiked prior to extraction, and extracted negative control samples spiked with the compounds after extraction were used to determine the recovery and matrix effects at 10 pg/mg. 

### 4.5. LC-MS Analysis

An Agilent 1260 high-performance liquid chromatography system was coupled with a Thermo Q Exactive mass spectrometer was used to analyze methylphenidate over a 12.5 min run following a 20 µL injection. Methylphenidate and D9-clenbuterol were separated on an ACE 3 µm C18 2.1 mm × 100 mm column held at 30 °C using a reverse phase gradient with a flow rate of 0.4 mL/min. Water with 0.2% formic acid was mobile phase A, and acetonitrile with 0.2 % formic acid was mobile phase B. The mobile phase composition at the start of the method was 3% B held for 0.4 min and increased to 99% over 7.1 min, which was then held for 0.7 min, and then, the composition was returned to initial conditions. Positive mode electrospray ionization was used to introduce the compounds to the mass spectrometer, which were detected using parallel reaction monitoring (PRM) of the following precursor ions 234.14 and 286.14 *m*/*z* for methylphenidate and D9-clenbuterol, respectively. The source conditions for the MS were as follows: source temperature, 300 °C; spray voltage, 4000 V; sheath gas, 50 arb units; sweep gas, 0 arb units; auxiliary gas, 10 arb units; ion transfer tube, 300 °C. The PRM scan parameters were as follows: scan range of 50–255 *m*/*z* (methylphenidate) and 50–310 *m*/*z* (D9-clenbuterol); resolution setting of 17,500, stepped HCD setting of 30, 80 eV isolation width of 1.0 *m*/*z*, automated gain control of 2e5, and a maximum ion transfer time of 100 ms. The mass spectrometer was calibrated using a mixture of caffeine, MRFA peptide, Ultramark 1621, and n-butylamine in an acetonitrile/methanol/acetic solution as prepared by the manufacturer (Thermo Fisher, Rockford, IL, USA).

### 4.6. Data Analysis

The LC-MS system was controlled by XCalibur software (Thermo, San Jose, CA, USA) and processed using Qual and Quan Browser with a 2 mDa window. The following product ions (84.08078, 56.04948, and 91.05423 *m/z*) were monitored for methylphenidate with ion 84.08078 *m/z* used as the quantifying ion. The 169.0512 *m/z* ion was used for D9-clenbuterol. Statistical calculations were accomplished using Excel (Microsoft, Redmond, WA, USA).

## 5. Conclusions

A fit-for-purpose LC-HRMS method was validated to detect and confirm the presence of methylphenidate in equine hair samples. To the authors’ knowledge, this is the first report of detections of methylphenidate in hair samples using identification criteria applicable for anti-doping applications. Furthermore, the use of a stepped collision energy PRM scan on the Q-Exactive system allows for a highly sensitive method with sufficient qualitative ions necessary for definitive identification. 

## Figures and Tables

**Table 1:** Inter- and intra-day assay accuracy (% expected) and precision (relative standard deviation) were determined at low, mid, and high QC levels (*n* = 6/level).

QC Level	Nominal Concentration (pg/mg)	Intra-Day Accuracy ^1^	Inter-Day Accuracy ^1^	Intra-Day Precision ^1^	Inter-Day Precision ^1^
Low	3	90.0	93.9	3.76	6.64
Mid	10	95.3	82.6	5.96	16.3
High	30	98.6	99.0	5.05	18.7

^1^ Accuracy is expressed as percentage of expected concentration and precision is expressed as relative standard deviation.

**Table 2:** Comparison of procedures used in the extraction and instrumental analysis of methylphenidate in hair.

Compounds Monitored	Extraction Approach	Instrumental Analysis	Reported Limit of Detection	Ref.
Methylphenidate, Ritalinic Acid	Liquid ^1^	LC-MS/MS	0.5 pg/mg	[8]
Methylphenidate	Liquid–Liquid ^2^	LC-MS/MS	30 pg/mg	[12]
Methylphenidate	Solid Phase ^3^	LC-MS	50 pg/mg	[11]
Methylphenidate	Liquid–Liquid ^4^	GC-MS	20 pg/mg	[10]

^1^ Methanol was the solvent. ^2^ Dichloromethane was the solvent. ^3^ Bond Elut Certify cartridge. ^4^ Isohexane in basic conditions was the solvent.

## Data Availability

The data presented in this study are available on request from the corresponding author. Some aspects of the data are not publicly available due to client confidentiality requirements.

## Supplementary Materials

The following are available online, Figure S1: Representative calibration curve of methylphenidate spiked in hair using linear regression analysis with 1/x weighting.

## Sample Availability

Samples are unavailable from the authors due to contractual requirements.
