# Supplementary material for: Detection of Methylphenidate in Equine Hair Using Liquid Chromatography–High-Resolution Mass Spectrometry
Source: Molecules. 2021 Sep 24;26(19):5798. doi: 10.3390/molecules26195798 (PMC8510229; doi:10.3390/molecules26195798)

molecules

MDPI

Supplementary Materials

Detection of Methylphenidate in Equine Hair Using Liquid Chromatography–High-Resolution Mass Spectrometry

Benjamin C. Moeller [1,2,*], Luis Flores [1], Amel Clifford [1], Gwendolyne Alarcio [1], Mary Mosburg [1] and Rick M. Arthur [3]

1 KL Maddy Equine Analytical Chemistry Laboratory, School of Veterinary Medicine, University of California, Davis, Davis, 95616 CA, USA; laflore@ucdavis.edu (L.F.); amlclifford@ucdavis.edu (A.C.); gsgonzales@ucdavis.edu (G.A.); mmmosburg@ucdavis.edu (M.M.)

2 Department of Molecular Biosciences, School of Veterinary Medicine, University of California, Davis, Davis, 95616 CA, USA

3 School of Veterinary Medicine, University of California, Davis, Davis, 95616 CA, USA; rmarthur@ucdavis.edu

* Correspondence: bcmoeller@ucdavis.edu; Tel.: +1-530-752-8700

Table of Contents	Page
**Figure S1.** Representative calibration curve of methylphenidate spiked in hair using linear regression analysis with 1/x weighting.	2

Figure S1. Representative calibration curve of methylphenidate spiked in hair using linear regression analysis with 1/x weighting.

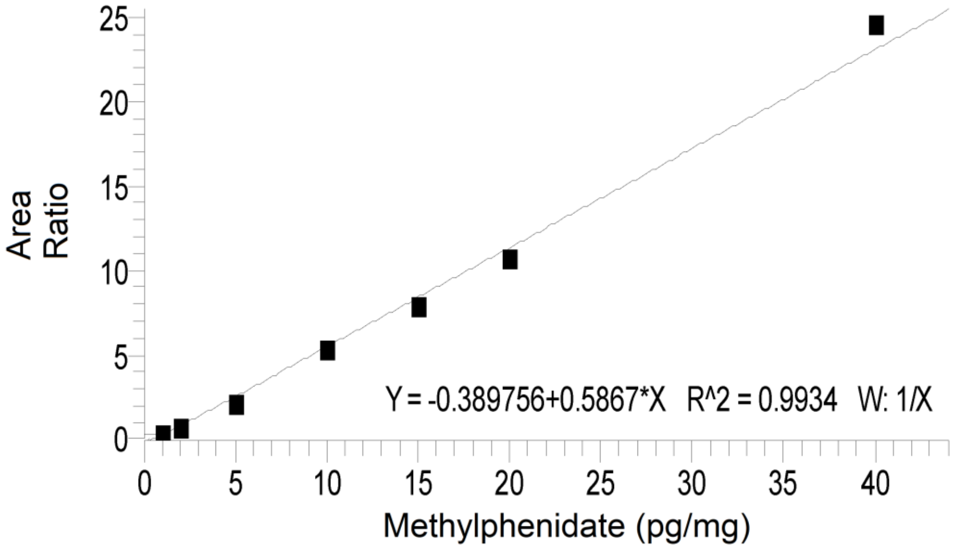

Supplement: Supplementary file 1 [file molecules-26-05798-s001.zip › molecules-1368276-supplementary.pdf]
